# One-Step Electrochemical Sensing of CA-125 Using Onion Oil-Based Novel Organohydrogels as the Matrices

**DOI:** 10.1021/acsomega.3c09149

**Published:** 2024-04-08

**Authors:** Omer Faruk Er, Hilal Kivrak, Duygu Alpaslan, Tuba Ersen Dudu

**Affiliations:** † Rare Earth Elements Research Institute, Turkish Energy Nuclear and Mineral Research Agency, Ankara 06980, Turkey; ‡ Department of Chemical Engineering, Faculty of Engineering, Van Yuzuncu Yil University, Van 65000, Turkey; § Department of Chemical Engineering, Faculty of Engineering and Architectural Sciences, Eskisehir Osmangazi University, Eskisehir 26040, Turkey; ∥ Translational Medicine Research and Clinical Center, Eskisehir Osmangazi University, Eskisehir 26040, Turkey

## Abstract

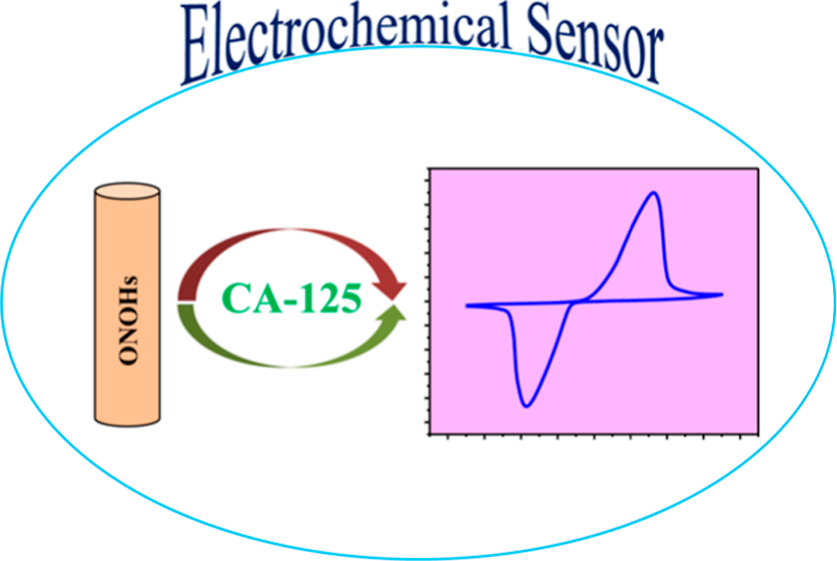

To reduce the high mortality rates caused by ovarian cancer, creating high-sensitivity, quick, basic, and inexpensive methods for following cancer antigen 125 (CA-125) levels in blood tests is of extraordinary significance. CA-125 is known as the exclusive glycoprotein employed in clinical examinations to monitor and diagnose ovarian cancer and detect its relapses as a tumor marker. Elevated concentrations of this antigen are linked to the occurrence of ovarian cancer. Herein, we designed organohydrogels (ONOHs) for identifying the level of CA-125 antigen at fast and high sensitivity with electrochemical strategies in a serum medium. The ONOH structures are synthesized with glycerol, agar, and glutaraldehyde and at distinct ratios of onion oil, and then, the ONOHs are characterized with Fourier transform infrared spectroscopy (FTIR) and scanning electron microscope (SEM). Electrochemical measurements are performed by cyclic voltammetry (CV), differential pulse voltammetry (DPV), and electrochemical impedance spectroscopy (EIS) in the absence and presence of CA-125 on the designed ONOHs. For the prepared ONOH-3 electrode, two distinct linear ranges are determined as 0.41–8.3 and 8.3–249.0 U/mL. The limit of quantitation and limit of detection values are calculated as 2.415 and 0.805 μU/mL, respectively, (S/N = 3). These results prove that the developed electrode material has high sensitivity, stability, and selectivity for the detection of the CA-125 antigen. In addition, this study can be reasonable for the practical detection of CA125 in serum, permitting early cancer diagnostics and convenient treatment.

## 1 Introduction

Tumor markers are structures produced by cancer tumors themselves or as a response to cancer in the presence of cancer by the body. In addition, these structures could be produced against neoplastic conditions like inflammation. Tumor markers containing hormones and several groups of glycoproteins such as enzymes, receptors, and oncofetal antigens could be found in tissues and various body fluids like blood and urine.^1−4^ Recently, the detection of tumor markers in the blood has become an important subject in cancer research, particularly in monitoring the condition during and after treatment, as well as evaluating the diagnosis and treatment of cancer patients.^5^ Tumor markers are used for the early diagnosis of cancer in asymptomatic patients.^6^ Different methods such as chemiluminescence,^7,8^ enzyme-linked immunosorbent assay (ELISA),^9^ mass spectrometry,^10^ array-based optical liquid-crystal (LC) immunodetection,^11^ fluorescence,^12^ and immunoradiometric assay^13^ have been used to detect tumor markers. However, these methods are time-consuming, expensive, labor-intensive, complex, tedious, and not appropriate for nonpoint-of-care applications. Therefore, detecting tumor markers is vital to the development and application of high-sensitivity, fast, and inexpensive detection methods.^14−16^ Electrochemical sensors (ESs) are of important interest due to their superior features such as rapid return, low cost, simplicity, easy portability, miniaturization, and high sensitivity.^17,18^

Ovarian cancer is the leading reason for death among female diseases due to its metastasis and recurrence depending on the late diagnosis.^19^ Almost all malignant and benign ovarian tumors emerge from one of the stromal, germ, and epithelial cells.^20^ When ovarian cancer is detected early, it can be treated with surgery followed by nonplatinum and platinum chemotherapy.^21^ Cancer antigen 125 (CA-125) is a submember of the MUCIN 16 family, which is used as a tumor marker of ovarian cancer and could be found at levels between 0.0 and 35.0 U/mL in blood samples.^22^ Different materials such as cacao oil-based organohydrogels (ONOHs),^23^ polyanthranilic acid (PAA)-modified glassy carbon screen-printed electrode (GSPE),^24^ sweet almond oil-based ONOHs,^25^ ZnO nanorod-Au nanoparticle (NP) nanohybrids,^26^ AuNPs/screen-printed gold electrode (SPGE),^27^ benzothiophene derivatives,^28−30^ and nonimprinted gold nanoelectrode ensemble (GNEE)^31^ have been used to increase the sensitivity of ES against CA-125. Further, Torati et al. reported that an ES was developed by using the Au nanostructure-modified electrode to detect CA-125. This sensor was found to indicate a good response to detect CA-125 with a 10–100 U/mL concentration range and 5.5 U/mL low detection limit values.^32^ In another study, Zheng et al. developed an ES by employing Prussian blue-platinum nanoparticles (PB-PtNPs). These PB-PtNPs were incorporated into a polyaniline (PANI) hydrogel to obtain PB-PtNPs-PANI and further enhance the signal. In order to further improve electrical conductivity and immobilize antibody, gold nanoparticles (AuNPs) were deposited on the surface of the PB-PtNPs-PANI hydrogel (Au-PB-PtNPs-PANI hydrogel) and were transferred on the glassy carbon electrode (GCE) to obtain PB-PtNPs-PANI hydrogel/GCE electrode. This electrode was found to exhibit high sensitivity for CA-125 with 4.4 mU/mL detection limit and a wide concentration range of 0.01–5000 U/mL.^33^

In this study, a novel approach was employed to detect the CA-125 cancer biomarker in serum medium using ESs with onion oil-based ONOHs. Different material-based ESs to detect CA-125 with high sensitivity and selectivity are reported in the literature. This study marks the first instance in the literature in which onion oil-based ONOHs were investigated for the detection of biomarkers with an ES. Hydrogels are structures that represent a large group of materials consisting of hydrophilic matrices that can absorb water at a high rate. The hydrogels offer great potential for healthcare and diagnostic applications due to their nontoxicity, biodegradability, and biocompatibility features.^34,35^ Hydrogels have been applied in many areas like separation matrices,^36^ enzyme carriers,^37^ and biomedical applications, which consist of drug delivery,^38^ biosensing,^39^ bone regeneration,^40^ immunotherapy,^41^ and tissue engineering.^42^ ONOHs are gels formed by physical or chemical cross-links of synthetic or naturally derived molecules, and these gels draw significant attention in drug delivery, nanopatterning, and photonics applications.^43−46^ These are synthesized by dispersing immiscible hydrophobic–hydrophilic polymer networks or hydrophilic polymer networks in a water–organic solvent system. These structures have great potential for smart material application areas due to their superior properties such as water retention and antifreezing, adjustable surface wettability, and solvent resistance. Herein, the synthesis of onion oil-based ONOHs was carried out using agar, glycerol, onion oil, and GA cross-linker through a free radical polymerization process. It is known that high concentrations of 1-methyl-2-propyldisulfane, 1,3-dipropyltrisulfane, and 3-((ethyltrisulfanyl)methyl)-3,4-dihydro-2*H*-thiopyran structures have been found in onion oil.^47^ The ES, which was developed using onion oil-based ONOHs without antibiomarkers, exhibited superior sensitivity, stability, and a wide linear range compared to those of the ESs documented in existing literature (Table 1).

**Table 1:** Performances of Distinct Electrode Systems Used to Detect CA-125 Compiled from Literature

biomarker	sensor	concentration range	detection limit	ref.
CA-125	Ag NPs-GQDs/Ab/BSA/Ag	0.01 U/mL	0.01–400 U/mL	(58)
CA-125	Co(bpy)_3_^3+^/MWNTs-Nafion/GC	1–30 U/mL 30–150 U/mL	0.36 U/mL	(59)
CA-125	FA@H-PANI@CS-HCl	0.25 pg/mL	0.001–25 ng/mL	(60)
CA-125	Ab_2_–Ag–Ab_1_/Au-VBG/BDD/Ta	0.09 mU/mL	0.5–100 U/mL	(61)
CA-125	MOF-808/CNT/GCE	0.001–0.1 ng/mL 0.1–30 ng/mL	0.5 pg/mL	(62)
CA-125	BSA/Ab/Au NPs/Cys A/ERGO-P(DA)-GCE	0.1 U/mL	0.1–400 U/mL	(63)
CA-125	Au-PB-PtNP-PANI hydrogel/GCE	0.01–5000 U/mL	4.4 mU/mL	(33)
CA-125	MPA/AuNPs@SiO_2_/QD/mAb	0–0.1 U/mL	0.0016 U/mL	(49)
CA-125	CuO nanoflakes	0.77–500 IU/mL	0.77 IU/mL	(64)
CA-125	ONOHs	0.41–8.3 U/mL	0.805 μU/mL	this study
		8.3–249 U/mL		

## 2 Materials and Methods

### 2.1 Materials

Chemicals like dopamine (98%), agar (99%), glutaraldehyde (GA) (50% in H_2_O), d-glucose (99.5%), methylene bis(acrylamide) (MBA) (99%), uric acid (≥99%), ethanol (≥99.8%), potassium chloride (KCl) (≥99%), glycerol (≥99%), acetone (≥99.9%), sodium hydrogen phosphate (Na_2_HPO_4_) (≥99%), calcium chloride (CaCl_2_) (≥97%), magnesium dichloride (MgCl_2_) (≥98%), ascorbic acid (99%), sodium chloride (NaCl) (≥99%), potassium hydrogen phosphate (K_2_HPO_4_) (98%), and potassium ferrocyanide (K_4_[Fe(CN)_6_]·3H_2_O) (≥98.5%) were used for the sensor designed and were supplied from Sigma-Aldrich. 0.9% isotonic sodium chloride solution was purchased from the local pharmacy. A potentiostat device (triple electrode system) that was used for measurements was purchased from CH Instruments. Deionized (DI) water that was used for measurements was obtained from a Milli-Q water purification system. All glassy materials were washed with DI water, ethanol, and acetone.

### 2.2 Electrochemical Measurements

**Scheme 1:**
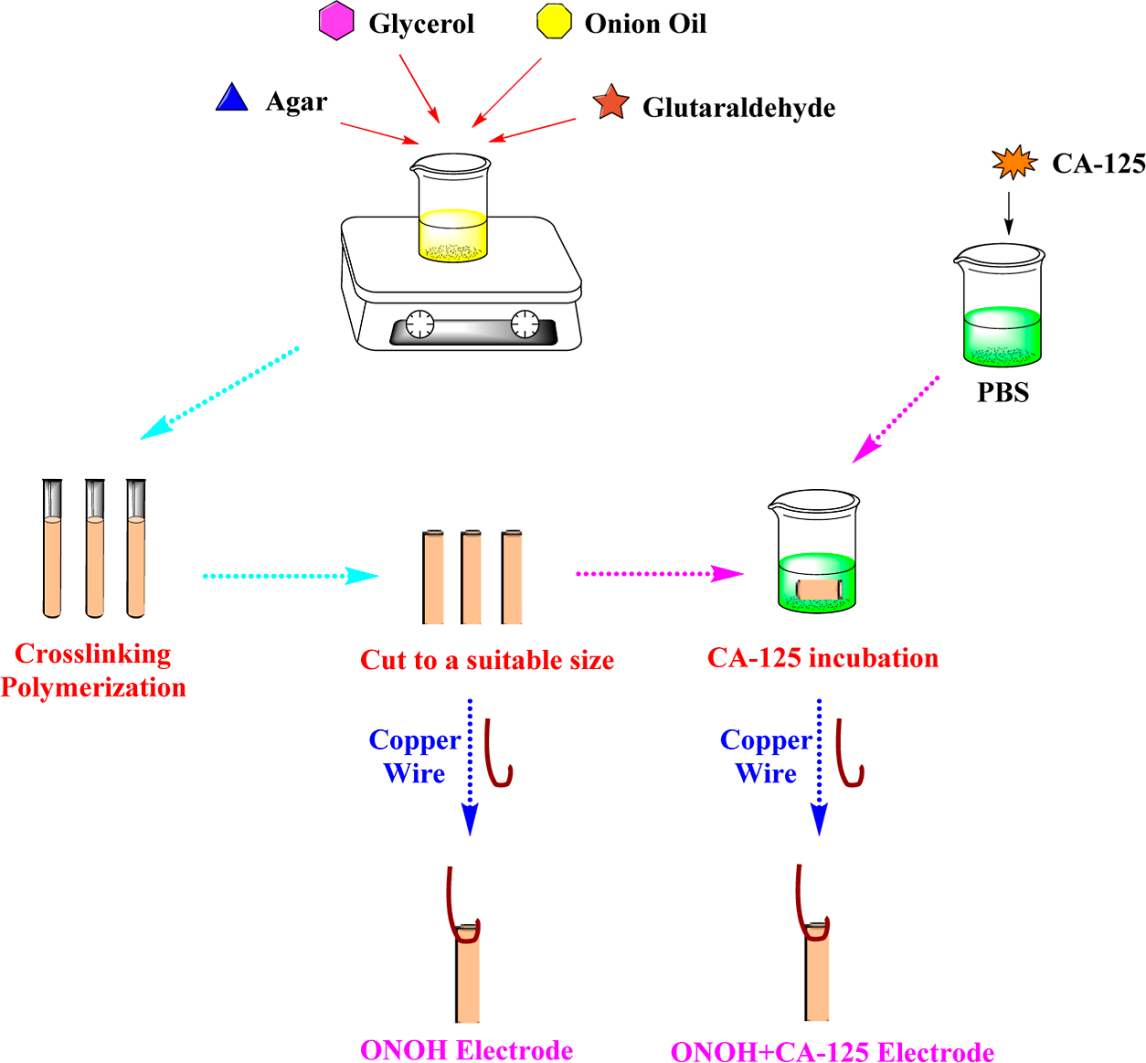
Preparation and Synthesis Steps of the ONOH Electrodes

The synthesized steps and characterization methods of the ONOH structures are presented in S1. The preparation steps of the ONOH electrode systems for electrochemical measurements are explained in S2. In addition, the steps of the ES preparation and synthesis are shown in Scheme 1.

Differential pulse voltammetry (DPV), cyclic voltammetry (CV), and electrochemical impedance spectroscopy (EIS) measurements were performed on the ONOHs prepared for the detection of CA-125, which is a tumor marker of ovarian cancer. Initially, CV measurements were performed with a scan rate of 50 mV/s in a pH: 7.4 PBS + 5.0 mM Fe(CN)_6_^3–/4–^ solution at room temperature with all ONOHs and ONOH + CA-125s obtained using CA-125 (500 ng/mL) at room temperature, and the results obtained were compared.

Second, to investigate the effect of the amount of CA-125 on the surface of ONOH-3, CV measurements were performed (scan rate: 50 mV/s) in a pH: 7.4 PBS + 5.0 mM Fe(CN)_6_^3–/4–^ solution. CV measurements were obtained over ONOH-3 + CA-125 generated with CA-125 amount among 1–5000 ng/mL at room temperature for 30 min incubation time. The 500 ng/mL concentration was found to be the best concentration. After determining the best concentration ratio, the effect of incubation time was studied with a scan rate of 50 mV/s in a pH: 7.4 phosphate-buffered saline (PBS) + 5.0 mM Fe(CN)_6_^3–/4–^ solution on ONOH-3 + CA-125 obtained by using CA-125 (500 ng/mL) at distinct incubation times of 10–110 min at room temperature. 30 min was found to be the best incubation time for preparing the ONOH-3 + CA-125 electrode. To understand the electro-oxidation process between CA-125 and ONOH-3, CV measurements were performed in a pH: 7.4 PBS + 5.0 mM Fe(CN)_6_^3–/4–^ solution at room temperature at distinct scan rates of 5–1000 mV/s over ONOH-3 + CA-125 obtained by using CA-125 (500 ng/mL) for 30 min incubation time.

To study load transfer resistance between CA-125 and the ONOH-3 electrode, EIS measurements were carried out in a pH: 7.4 PBS + 5.0 mM Fe(CN)_6_^3–/4–^ solution at room temperature and at distinct potentials between −0.6 and −0.5 V on ONOH-3 + CA-125 obtained by using CA-125 (500 ng/mL) for 30 min.

The sensitivity of ONOH-3 against CA-125 was determined with DPV measurements in a pH: 7.4 PBS + 5.0 mM Fe(CN)_6_^3–/4–^ solution at room temperature on ONOH-3s and ONOH-3 + CA-125s produced with distinct concentrations of CA-125 of 0.01–5000 ng/mL for 30 min.

To analyze the effect on the electro-oxidation process between CA-125 and ONOH-3 of structure molecules found in serum medium, CV (scan rate: 50 mV/s) and EIS (0.0 V) measurements were performed in 2.5 mM uric acid + pH: 7.4 PBS, 0.1 mM dopamine + pH: 7.4 PBS, 4.7 mM glucose + pH: 7.4 PBS, and 0.1 mM ascorbic acid + pH: 7.4 PBS over ONOH-3 and ONOH-3 + CA-125 produced by using CA-125 (500 ng/mL) for 30 min.

Finally, the effects of salts found in serum medium on the electro-oxidation process between ONOH-3 and CA-125 were studied with EIS (0.0 V) and CV (scan rate: 50 mV/s) in an artificial solution and a 0.9% isotonic sodium chloride solution over ONOH-3 + CA-125 produced with CA-125 (500 ng/mL) for 30 min. An artificial serum was prepared with d-glucose (4.7 mM), uric acid (2.5 mM), CaCl_2_ (5.0 mM), KCl (4.5 mM), and MgCl_2_ (1.6 mM).

## 3 Results and Discussion

**Figure 1:**
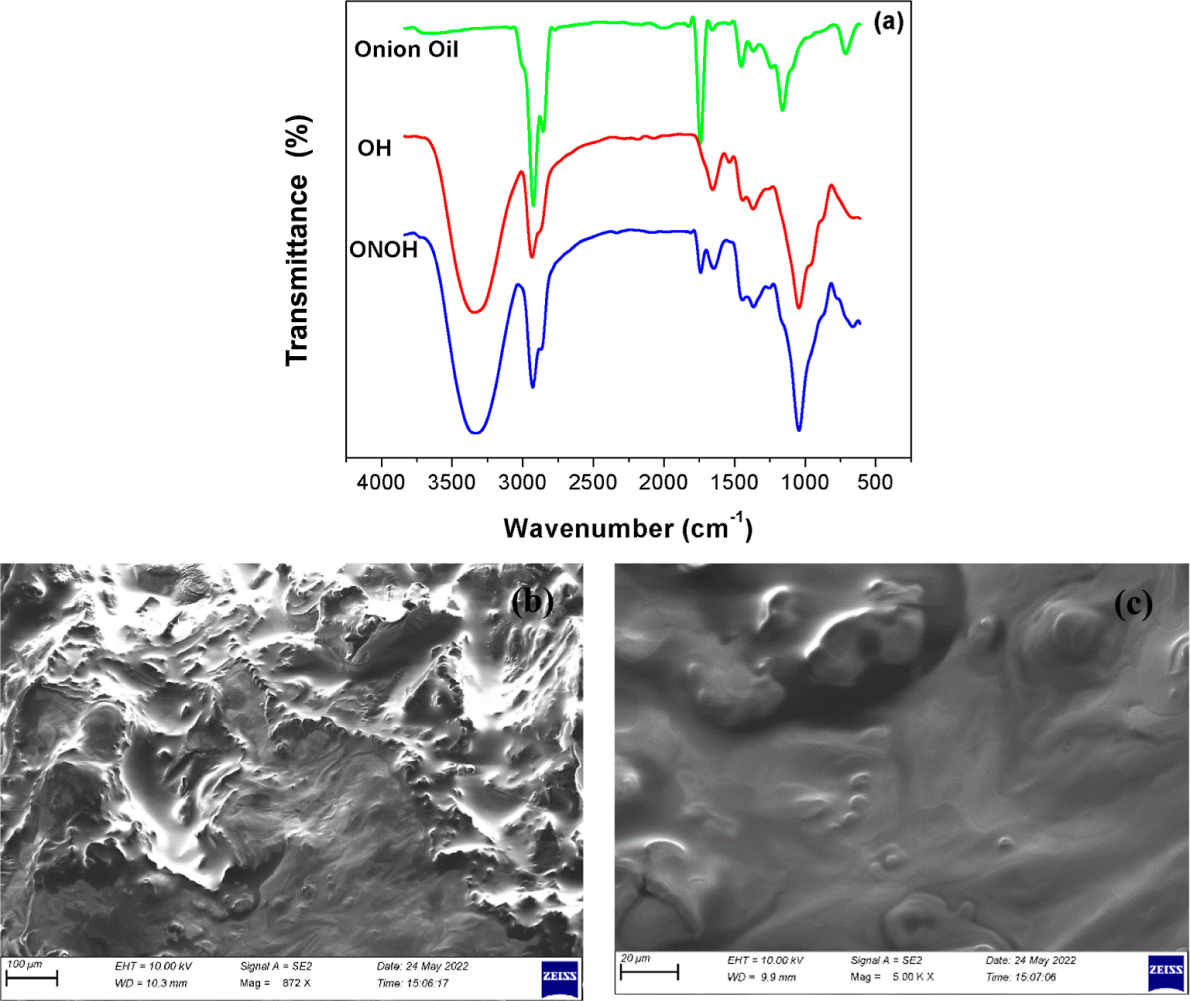
(a) FT-IR spectra and (b,c) SEM images of the onion oil-based ONOH.

The developed ONOH structure was characterized by FT-IR and scanning electron microscopy (SEM). The FT-IR spectra of onion oil, ONOH (OH), and onion oil-based ONOH structures are given in Figure 1a. The peaks of onion oil were observed as strong and moderate on average at 2930 cm^–1^ (C–H bonds), 2860 cm^–1^ (C–H bonds), 1740 cm^–1^ (C=O and C–O bonds), 1440 cm^–1^ (C–H bonds), 1170 cm^–1^ (C=O and C–O bonds), and 720 cm^–1^ (C=C bonds). Among these peak intensities, those at 2930, 2860, 1740, 1440, 1170, and 720 cm^–1^ are seen as low intensities in the spectrum of the ONOH structure. In addition, peak intensities at 3310 cm^–1^ (O–H bonds), 2930 cm^–1^ (C–H bonds), 1440 cm^–1^ (C–H bonds), and 1030 cm^–1^ (C=O and C–O bonds) cm^–1^ due to the ONOH structure can be observed. These peaks appeared with higher intensity in comparison to those of the OH structures. It can be stated that after onion oil was incorporated into the OH network, bands from distinctive aromatic compounds became visible. The peaks observed at 3310, 2930, and 1030 cm^–1^ in the ONOH structure have deepened or widened. These changes occurring on the characteristic peaks indicate that the ONOH structures were successfully synthesized with onion oil.

The surface morphology analysis of onion oil-based ONOH was performed with an SEM device. SEM images of the ONOH structure are given in Figure 1b,c. It is seen that the oil globules of onion oil in the ONOH structure penetrated well toward the surface. It can be seen that the onion oil globules have a homogeneous distribution on the ONOH surface. The ONOH surface was observed to be flat with a background caused by onion oil.

**Figure 2:**
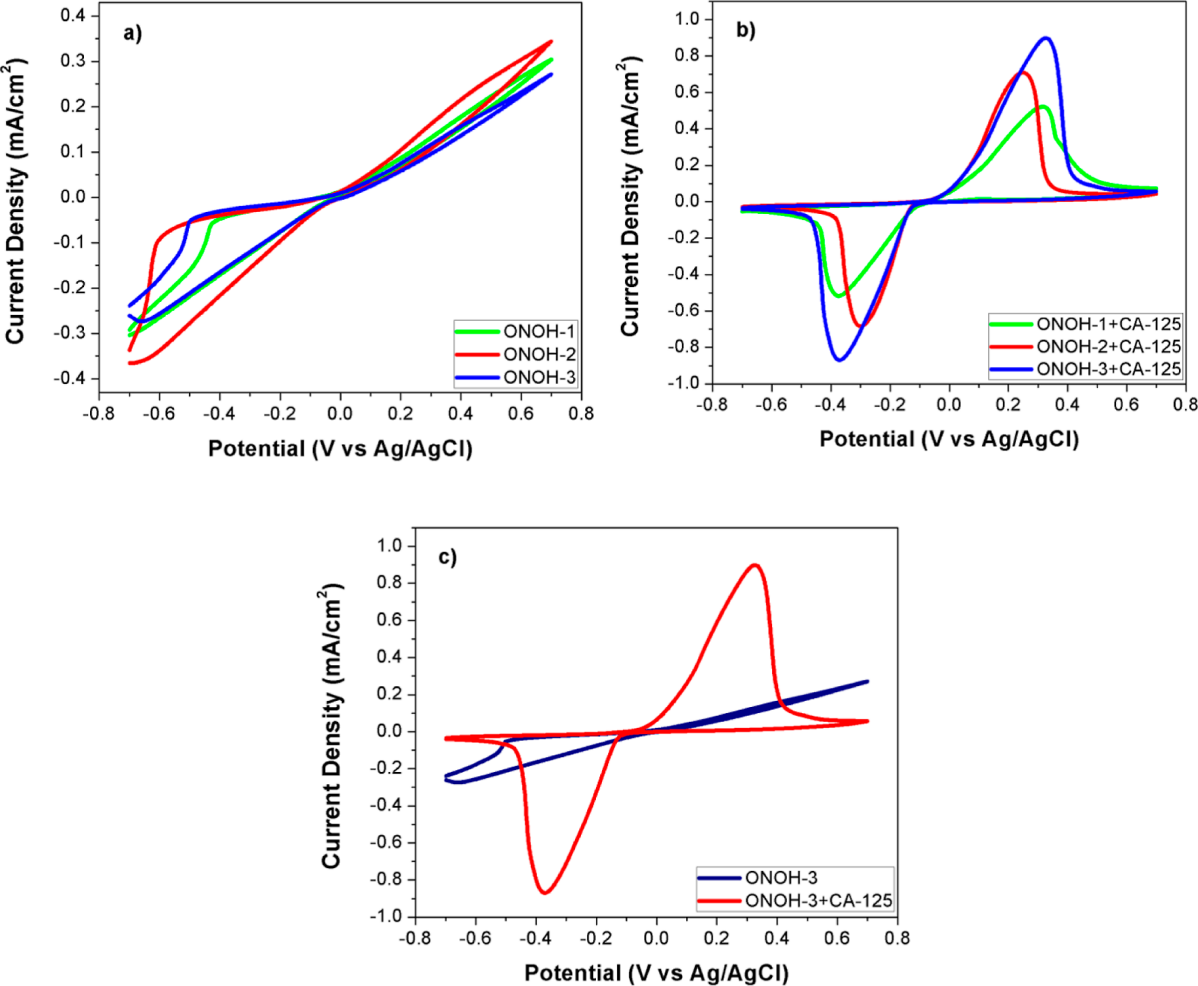
CV results on (a) ONOHs without CA-125, (b) ONOH + CA-125 by using CA-125 (500 ng/mL) for 30 min in a pH: 7.4 PBS + 5.0 mM Fe(CN)_6_^3–/4–^ solution at room temperature and 50 mV/s scan rate, and (c) comparison of ONOH-3 and ONOH-3 + CA-125.

Electrochemical measurements of the synthesized ONOHs were performed using DPV, EIS, and CV techniques to detect the CA-125. CV measurements, initially, on ONOHs were obtained at potentials between −0.7 and 0.7 V and room temperature (scan rate: 50 mV/s). All results are presented in Figure 2. No oxidation peaks were observed in the measurements obtained without CA-125. In addition, among the synthesized ONOHs, ONOH-2 had the best activity in terms of total potential (Figure 2a). Measurements with ONOH + CA-125 obtained by incubation (30 min) with CA-125 (500 ng/mL) showed forward and backward peaks between 0.0 and 0.6 potentials. These peaks are electro-oxidation peaks belonging to CA-125 (Figure 2b). ONOH-3 + CA-125 displayed the highest activity with 0.9096 mA/cm^2^ (909.6 μ/cm^2^) at a 0.33 potential of forward peak and 0.8761 mA/cm^2^ (876.1 μ/cm^2^) at a −0.37 potential of backward peak values (Figure 2c). These values were found to be very promising results according to the studies reported in the literature.^48,49^ Moreover, ONOH-1 + CA-125 showed the lowest performance with 0.5304 mA/cm^2^ (530.4 μ/cm^2^) at a 0.31 potential of forward peak and 0.5291 mA/cm^2^ (529.1 μ/cm^2^) at a −0.37 potential of backward peak values (Figure 2b). It can be noted that these results are quite interesting for the detection of CA-125.

**Figure 3:**
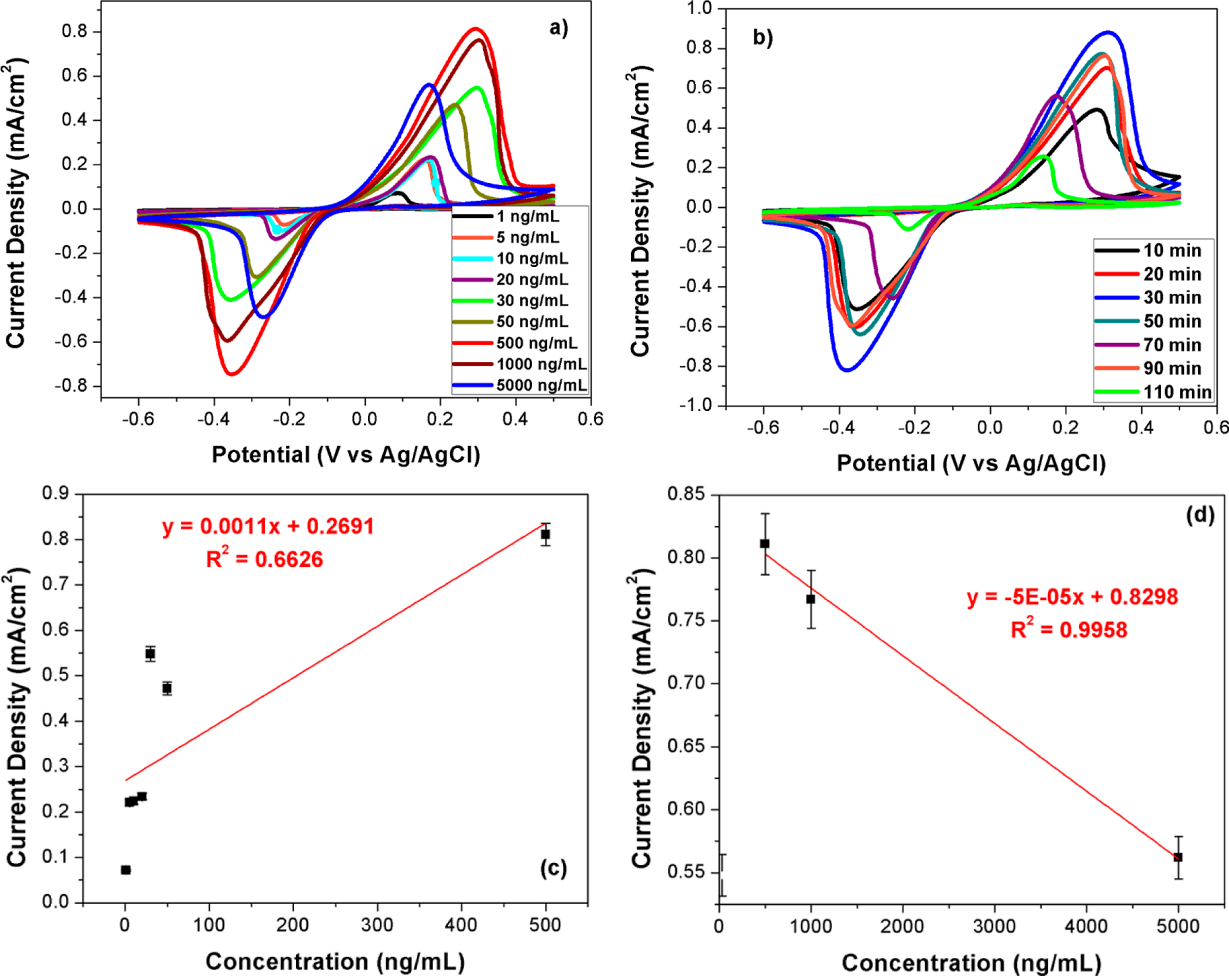
CV results on (a) ONOH-3 + CA-125 produced with distinct CA-125 concentrations of 1–5000 ng/mL for 30 min, (b) ONOH-3 + CA-125 produced with CA-125 (500 ng/mL) for distinct incubation times (10–110 min) at room temperature in a pH: 7.4 PBS + 5.0 mM Fe(CN)_6_^3–/4–^ solution (scan rate: 50 mV/s), and (c,d) current density and concentration plots from distinct CA-125 concentrations of 1–5000 ng/mL.

After determining that ONOH-3 shows the best performance among the ONOHs prepared, measurements were performed over ONOH-3 + CA-125 for optimum concentration and incubation time. Measurements were taken with the CV technique at a 50 mV/s scan rate between −0.6 and 0.5 potentials over ONOH-3 + CA-125 obtained by using CA-125 (1–5000 ng/mL) for 30 min. The results are given in Figure 3a. The electro-oxidation peaks among 0.0–0.6 potentials were observed for all concentration ratios. A regular increase from 1 to 500 ng/mL (Figure 3c) and a regular decrease from 500 to 5000 ng/mL (Figure 3d) in maximum current density were observed. The maximum current density was obtained for a 500 ng/mL CA-125 concentration. To study the incubation time effect, CV measurements were performed with a scan rate: 50 mV/s between −0.6 and −0.5 potentials over ONOH-3 + CA-125 obtained by using CA-125 (500 ng/mL) for a distinct incubation time of 10–110 min at room temperature. Results are presented in Figure 3b. A regular increase in current density between 10 and 30 min and a regular decrease between 30 and 110 min were observed. The maximum current density was seen for ONOH-3 + CA-125 prepared with a 30 min incubation time.

**Figure 4:**
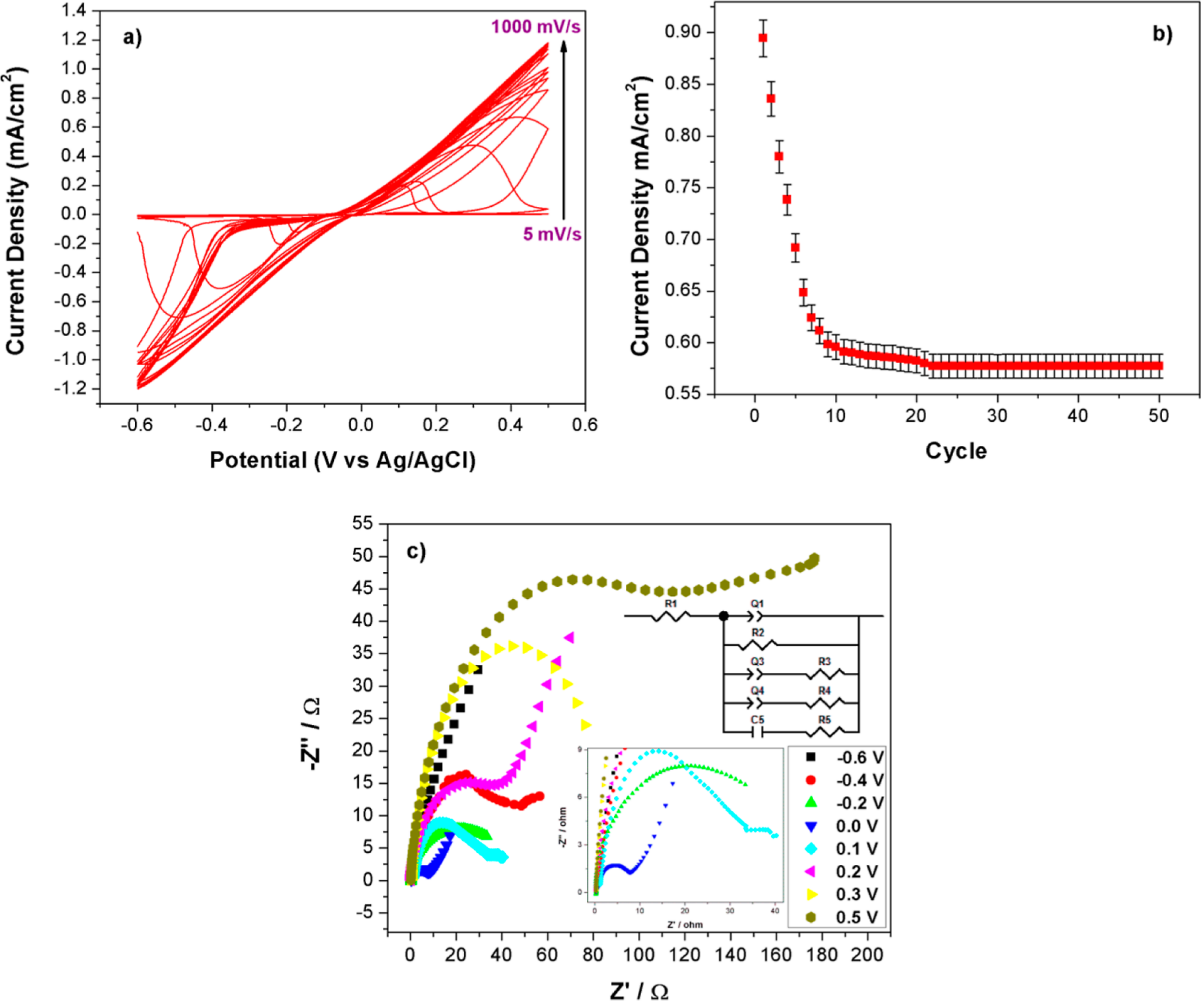
(a) CV results at distinct scan rates (5–1000 mV/s) in the pH: 7.4 PBS + 5.0 mM Fe(CN)_6_^3–/4–^ solution on ONOH-3 + CA-125 obtained by using CA-125 (500 ng/mL) for 30 min, (b) CV stability in the 50 cycles of the ONOH-3 + CA-125 electrode, and (c) Nyquist plots at distinct potentials of −0.6–0.5 in the pH: 7.4 PBS + 5.0 mM Fe(CN)_6_^3–/4–^ solution on ONOH-3 + CA-125 obtained by using CA-125 (500 ng/mL) for 30 min at room temperature.

To analyze the electro-oxidation process between ONOH-3 and CA-125, EIS and CV measurements were performed. CV measurements were performed over ONOH-3 + CA-125s obtained by using CA-125 (500 ng/mL) for 30 min at the distinct scan rates (5–1000 mV/s) and −0.6–0.5 potentials. Results are shown in Figure 4a. A regular increase in maximum current density was observed from 5 to 1000 mV/s. This event shows that a diffusion-controlled electrochemical reaction occurs on the surface of ONOH-3. The stability of ONOH-3 + CA-125 was researched on the 50 cycles with CV in the pH: 7.4 PBS + 5.0 mM Fe(CN)_6_^3–/4–^ solution from −0.6 to 0.5 potentials, and the results are given in Figure 4b. It was observed that the CV stability of ONOH-3 + CA-125 showed a rapid decrease in the first eight cycles and then stabilized after the eighth cycle. These results prove that the ONOH-3 + CA-125 electrode has high stability and repeatability properties.

EIS measurements were performed at 5 mV amplitude, and distinct potentials between −0.6 and 0.5 V over ONOH-3 + CA-125 were obtained by using CA-125 (500 ng/mL) for 30 min at room temperature. EIS is a technique that is frequently used in analyzing materials in areas such as biology, electrochemistry, medicine, material science, and sensor. The Nyquist plots obtained from EIS data take place at a linear cross-section and a semicircular area expressing a diffusion-controlled reaction and load transfer resistance (*R*ct) on the material surface.^50−54^ The Nyquist plots are given in Figure 4c. A gradual decrease between −0.6 (59.82 Ω) and 0.0 (9.55 Ω) potentials and a gradual increase between 0.0 (9.55 Ω) and 0.5 (75.23 Ω) potentials were observed in the semicircular area. When the diameter of these semicircles is large, the charge transfer resistance is large, and when it is small, the charge transfer resistance is small. The small charge transfer resistance is expressed that occurs fast oxidation kinetics of CA-125 antigen electro-oxidation over the surface of ONOH-3.^47,55−57^ The lowest charge transfer resistance was obtained over a potential of 0.0 (9.55 Ω), and this potential can be the onset potential for the electro-oxidation of the CA-125 antigen. Moreover, at 0.0 potential, both the semicircle expressing the electron transfer and the linear part expressing the diffusion-controlled reaction were observed, and these results support the results found in the scan rate study (Figure 4a–c).

**Figure 5:**
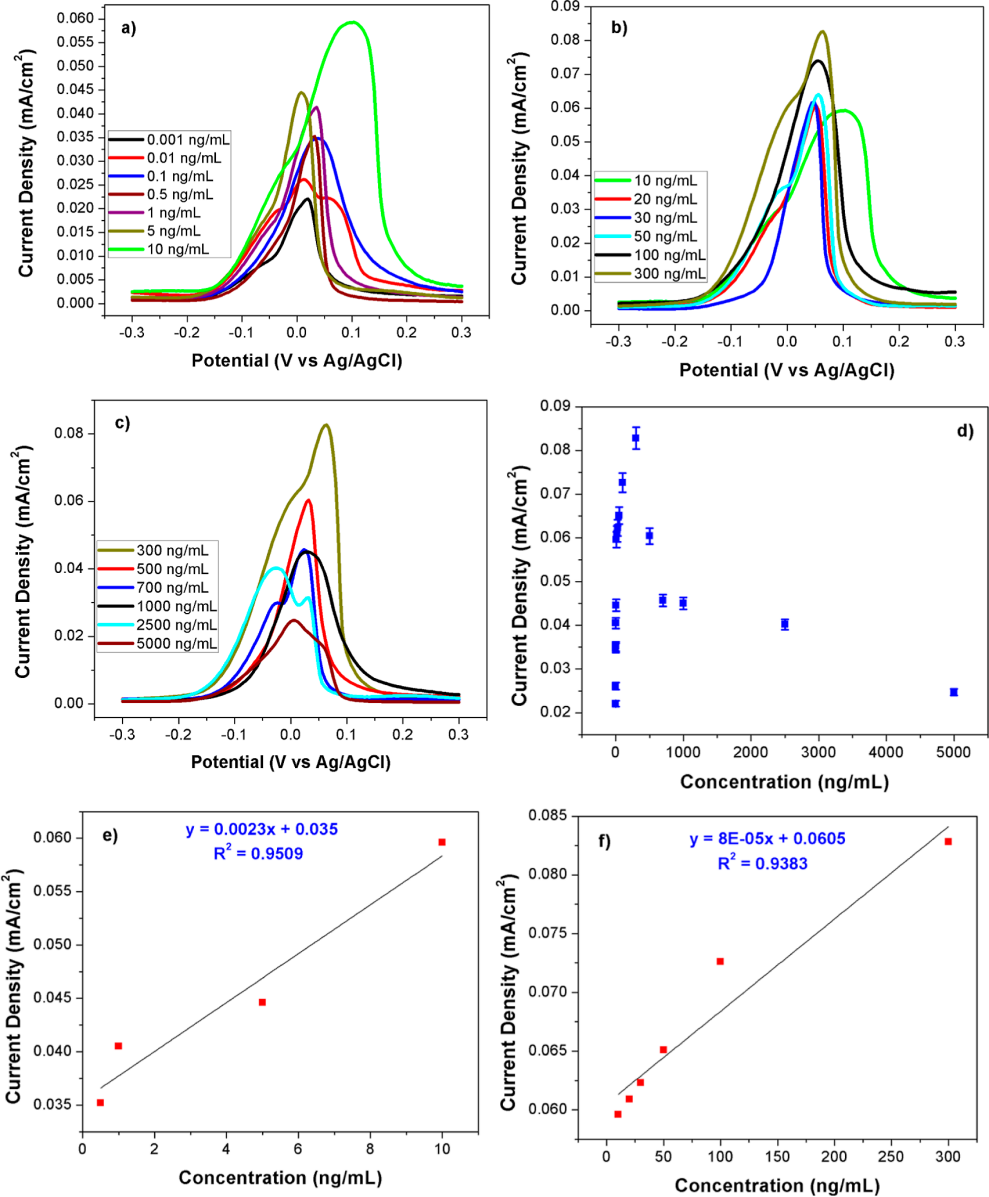
DPV results in the pH: 7.4 PBS + 5.0 mM Fe(CN)_6_^3–/4–^ solution over ONOH-3 + CA-125 obtained by using distinct CA-125 at (a–c) 0.001–5000 ng/mL for 30 min, and (d–f) concentrations vs maximum current densities.

Features that determine the sensitivity such as limit of detection (LOD) and limit of quantification (LOQ) of the ES were researched via DPV in a pH: 7.4 PBS + 5.0 mM Fe(CN)_6_^3–/4–^ solution over ONOH-3 + CA-125 obtained by using varying CA-125 amounts of 0.01–5000 ng/mL for 30 min at room temperature. All DPV results and maximum current densities vs concentrations plots are presented in Figure 5. A linear increase in maximum current densities was observed for concentrations between 0.01 and 500 ng/mL, and a linear decrease in maximum current densities was observed for concentrations between 500 and 5000 ng/mL (Figure 5a–d). It was found that the sensor works in two different linear ranges as follows: 0.5–10 and 10–300 ng/mL (Figure 5a,b). *R*^2^s for these two different linear ranges are designated as 0.9509 and 0.9383 (Figure 5e,f), respectively. From all these data, LOD and LOQ values were calculated as 0.805 and 2.415 μU/mL, respectively. These values were found to be lower than the values of the sensors reported in the literature for the detection of CA-125 (Table 1).

**Figure 6:**
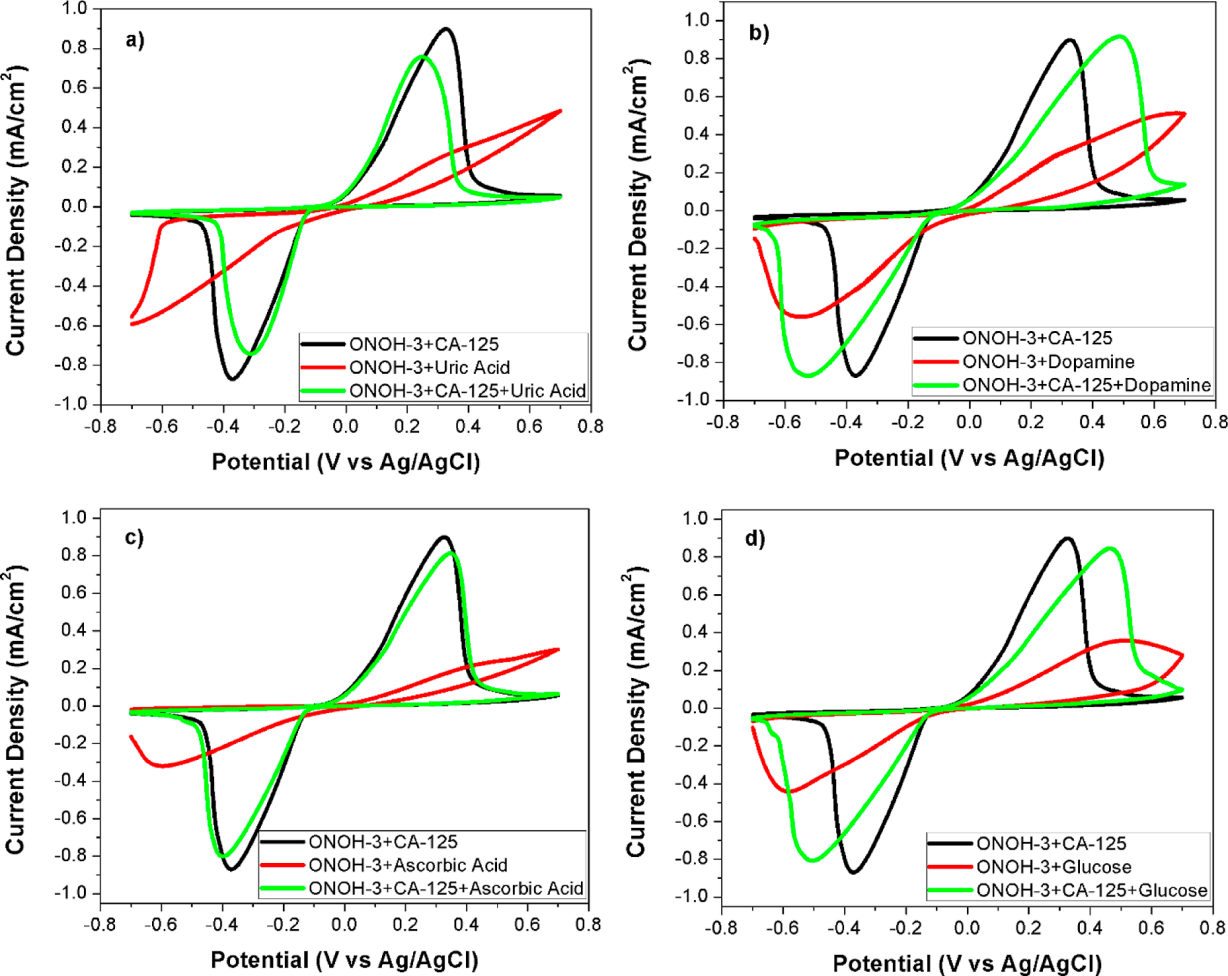
CV results in (a) PBS + uric acid, (b) PBS + dopamine, (c) PBS + ascorbic acid, and (d) PBS + glucose at 50 mV/s scan rate and room temperature on ONOH-3 and ONOH-3 + CA-125 obtained by using CA-125 (500 ng/mL) for 30 min.

**Figure 7:**
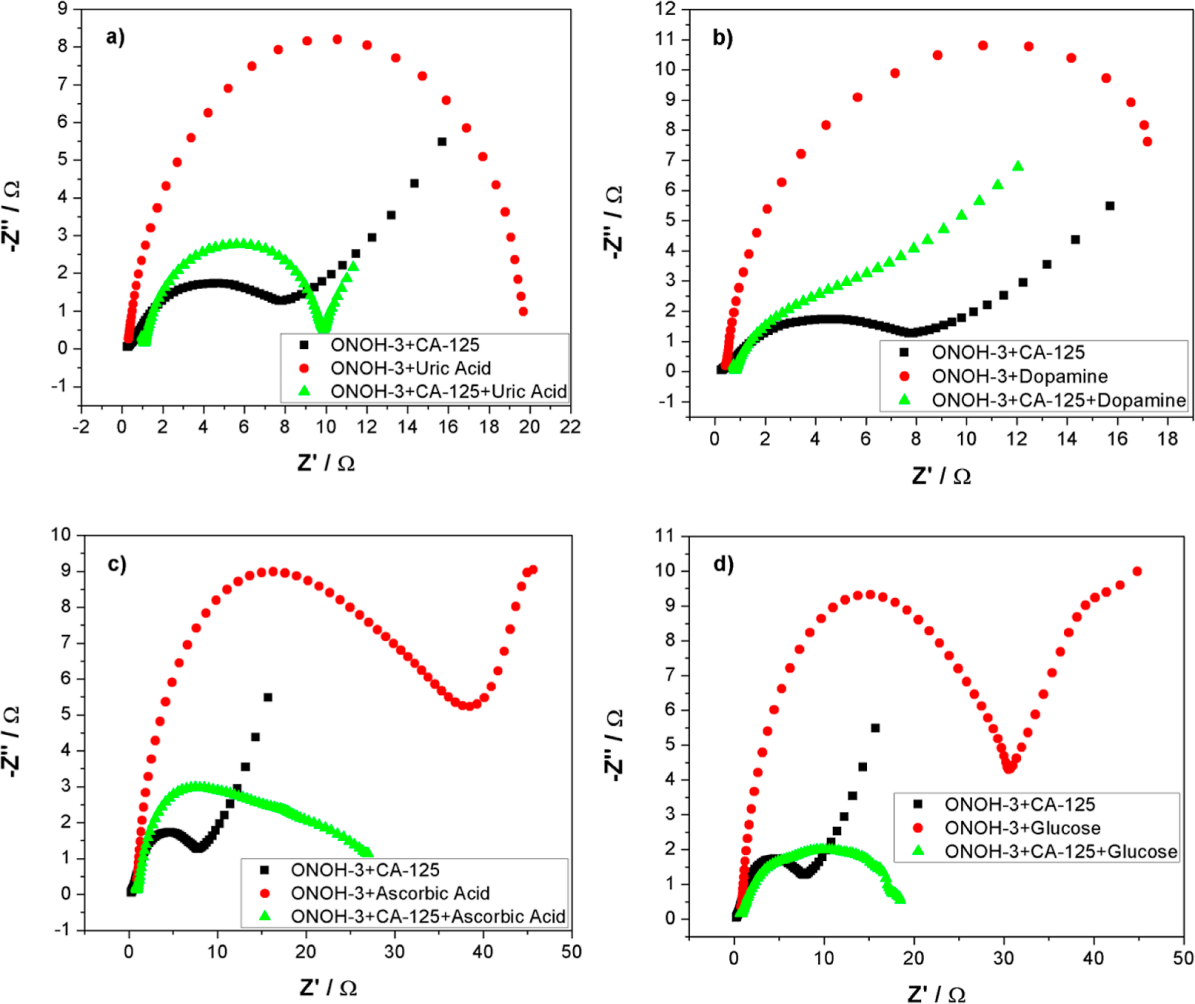
Nyquist plots obtained from ESI measurements at 0.0 V in (a) PBS + uric acid, (b) PBS + dopamine, (c) PBS + ascorbic acid, and (d) PBS + glucose at room temperature on ONOH-3 and ONOH-3 + CA-125 obtained by using CA-125 (500 ng/mL) for 30 min.

To study the interference effect on the electro-oxidation reaction between CA-125 and ONOH-3 of structure molecules like uric acid, glucose, ascorbic acid, and dopamine that were found in serum medium, EIS and CV measurements were performed. CV results (scan rate: 50 mV/s) and EIS data (5 mV amplitude and 0.0 V) were obtained over ONOH-3 + CA-125 obtained using CA-125 (500 ng/mL) for 30 min and in pH: 7.4 PBS + 2.5 mM uric acid, pH: 7.4 PBS + 0.1 mM dopamine, pH: 7.4 PBS + 4.7 mM glucose, and pH: 7.4 PBS + 0.1 mM ascorbic acid solutions. CV results and the Nyquist plots are presented in Figures 6 and 7, respectively. In Figure 6, it can be seen that the effects of ascorbic acid, uric acid, glucose, and dopamine structures on the electro-oxidation process were very low between ONOH-3 and CA-125. At the same time, when measurements were performed over ONOH-3 without CA-125, no electro-oxidation peaks were observed (Figure 6). However, it can be seen that this caused a potentially small shift upward in the maximum current density of glucose and dopamine structures (Figure 6b–d). Conversely, it was found that the uric acid structure causes a slight downward area shift as the potential on the maximum current density (Figure 6a). Among these structures, it was observed that ascorbic acid has the weakest effect on the oxidation between CA-125 and ONOH-3 (Figure 6c). In the Nyquist plots obtained over ONOH-3 without CA-125, high electron transfer resistances were observed that corresponded to the presence of CA-125 for all structure molecules. Data obtained over ONOH-3 + CA-125 were found to be close to each other(Figure 7). It can be clearly stated that the EIS results and CV results are harmonious.

**Figure 8:**
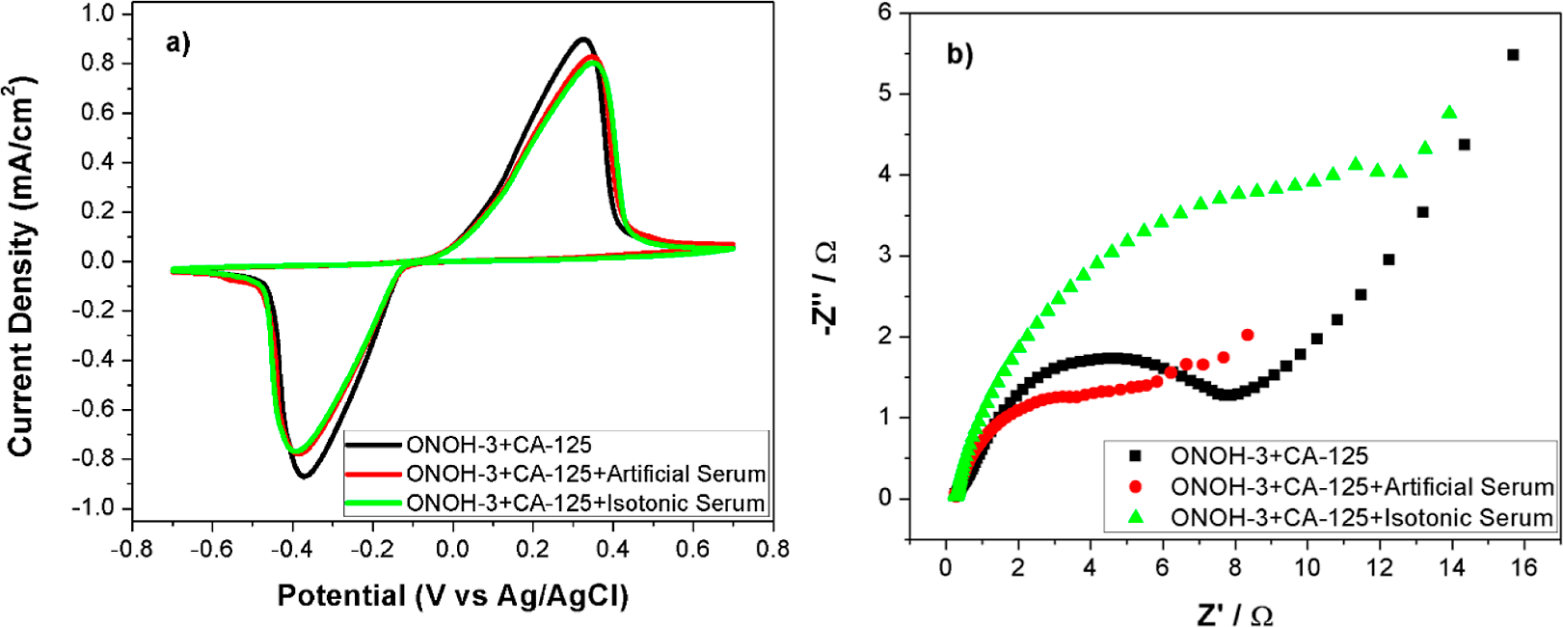
(a) CV results (scan rate: 50 mV/s) and (b) Nyquist at 0.0 potential in artificial and isotonic serums on ONOH-3 + CA-125 obtained by using CA-125 (500 ng/mL) for 30 min.

Finally, to investigate the effect on the electro-oxidation process between ONOH-3 and CA-125 of the salts found in the blood, measurements were carried out in artificial and isotonic serums with CV and EIS techniques over ONOH-3 + CA-125s obtained by using CA-125 (500 ng/mL) for 30 min. The results are shown in Figure 8. It may clearly be seen in CV results that no effects on the electro-oxidation of the salts exist (Figure 8a). In the same way, similar electron transfer resistances were also observed in the Nyquist plots (Figure 8b).

## 4 Conclusions

In this study, we improved an ES with onion oil-based novel ONOHs to detect CA-125 in a serum medium. The ONOHs were analyzed via distinct water–organic solutions, FT-IR, and SEM. The ES was designed by incubating CA-125 on the ONOHs without anti-CA-125. CV measurements were performed with the ES designed in the absence and presence of the CA-125 antigen. An incubation time of 30 min and a concentration of 500 ng/mL CA-125 were determined as optimal conditions for the designed sensor. The current densities directly proportional to the amount of onion oil in the ONOHs were observed. ONOH-3 among the ONOHs exhibited the highest performance with a maximum current density value of 0.9097 mA/cm^2^ under optimal conditions. Furthermore, the electron transfer resistance of ONOH-3 in the presence of CA-125 was found to be low in the absence of CA-125. The LOD and LOQ values of the sensor were determined to be 0.805 and 2.415 μU/mL, respectively, and the detection limits were found in two different linear ranges of 0.5–10 and 10–300 ng/mL. These results indicate that it has a very high sensitivity to CA-125 of ONOH-3. ONOH-3 was found to have a high selectivity to CA-125 despite interference effects of distinct structure molecules found in the serum medium. In addition, it was also seen that there was no effect of salts found in the serum medium of electrochemical reaction between ONOH-3 and CA-125 antigen when measurements were carried out in an artificial serum. Results show that onion oil embedded in the 3D network porous morphology of the ONOH increased the electrocatalytic activity and caused low charge transfer resistance in the CA-125 electro-oxidation reaction. This positive effect caused the designed onion oil-based ONOHs to exhibit high sensitivity, good stability, high selectivity, and low detection limits toward CA-125. As a result, these results prove that ONOH-3 has great hope for clinical applications of ovarian cancer due to its high sensitivity, selectivity, and stability against CA-125 antigen.

## Supporting Information Available

Synthesis of organo-hydrogels, production of the ES with ONOHs, and swelling test results of the ONOHs (PDF)
