# Supplementary material for: One-Step Electrochemical Sensing of CA-125 Using Onion Oil-Based Novel Organohydrogels as the Matrices
Source: ACS Omega. 2024 Apr 8;9(16):17919–30. doi: 10.1021/acsomega.3c09149 (PMC11044171; doi:10.1021/acsomega.3c09149)
Supplement: Supplementary file 1 — ao3c09149_si_001.pdf [file ao3c09149_si_001.pdf]

One-step electrochemical sensing of CA-125 using onion oil-based novel organohydrogels as the matrices

Omer Faruk Er[a,b], Hilal Kivrak[c,d*], Duygu Alpaslan[e], Tuba Ersen Dudu[e]

[a]*Rare Earth Elements Research Institute, Turkish Energy Nuclear and Mineral Research Agency, 06980, Ankara, Turkey*

[b]*Department of Chemical Engineering, Faculty of Engineering, Van Yuzuncu Yil University, 65000, Van, Turkey*

[c]*Department of Chemical Engineering, Faculty of Engineering and Architectural Sciences, Eskisehir Osmangazi University, Eskisehir, 26040, Turkey*

[d]*Translational Medicine Research and Clinical Center, Eskisehir Osmangazi University, 26040 Eskisehir, Turkey*

[e]*Department of Chemical Engineering, Faculty of Engineering, Van Yuzuncu Yil University, 65000, Van, Turkey*

*Corresponding Author: hilalkivrak@gmail.com

Supporting Information

S1. Characterization and Synthesis of Organo-hydrogels

The Onion Oil-based Organohydrogels (ONOHs) were synthesized as described by Alpaslan et al[3]. The 2 mL of agar solution and 0.04 mL of glycerol were added in a flask and different amounts 0.1 mL, 0.2 mL, and 0.3 mL onion oil were added to the reactions mixture (Table S2). The reaction mixture was stirred at 800 rpm for 15 min until the formation of a clear homogeneous solution emulsion and glutaraldehyde reagent was added as a crosslinker and further homogenized. The polymerization process was started by adding the initiator solution APS. The solution was pipetted into a 6 mm diameter tube and cut into 6 mm long cylinders after 4 hours. The oven was at 40 ^{0}C until a constant weight was achieved and stored at 4 ^{0}C for further uses.

For the analyses, the swelling analysis methods mentioned in the literature were applied[1, 2]. The Fourier Transform Infrared Spectroscopy was measured with a Fourier Transform

Infrared Spectrometer at a frequency range of 4000-650 cm^{-1} by weighing out 8-10 mg ONOHs.

Table S1. Contents of ONOHs prepared with onion oil

Mix	Onion Oil Amount (mL)	Cod	Cross-linker
Agar + Glycerol	0.1	ONOH-1	Glutaraldehyde (GA)
	0.2	ONOH-2	Glutaraldehyde (GA)
	0.3	ONOH-3	Glutaraldehyde (GA)

S2. Fabrication of the electrochemical sensor

The ONOHs prepared to detect CA-125 were cut into 1 cm size and CA-125 incubated over ONOH electrodes (0.5 cm the surface coverage area) at varying concentrations for certain periods. Finally, the copper wires were added to the end of the PORHs that were unincubated with CA-125 antigen. The surface coverage areas of the electrodes were calculated using the area formula of the cylinder. Electrochemical measurements like CV, EIS, and DPV were performed with potentiostat device that triple electrode system. The ONOHs were used as working electrode. The counter electrode and reference electrode were used as Pt wire and Ag/AgCl (3 M KCl), respectively.

S.3. Characterization Results

The change in S% of gel (AG), organohydrogel (p(AG-g)), and ONOH as a function of solvent concentration in water-organic solvent mixtures were shown in Figure S2. After the addition of onion oil to ONOHs, the ID water and tap water absorption capacity decreased ratio 14-58% and 15-65%, respectively. The quantity of essential oil and the swelling values were shown to have an inverse relationship. The S% of ONOHs in various organic solvent-water mixes has been discovered to be influenced by the solvent composition.

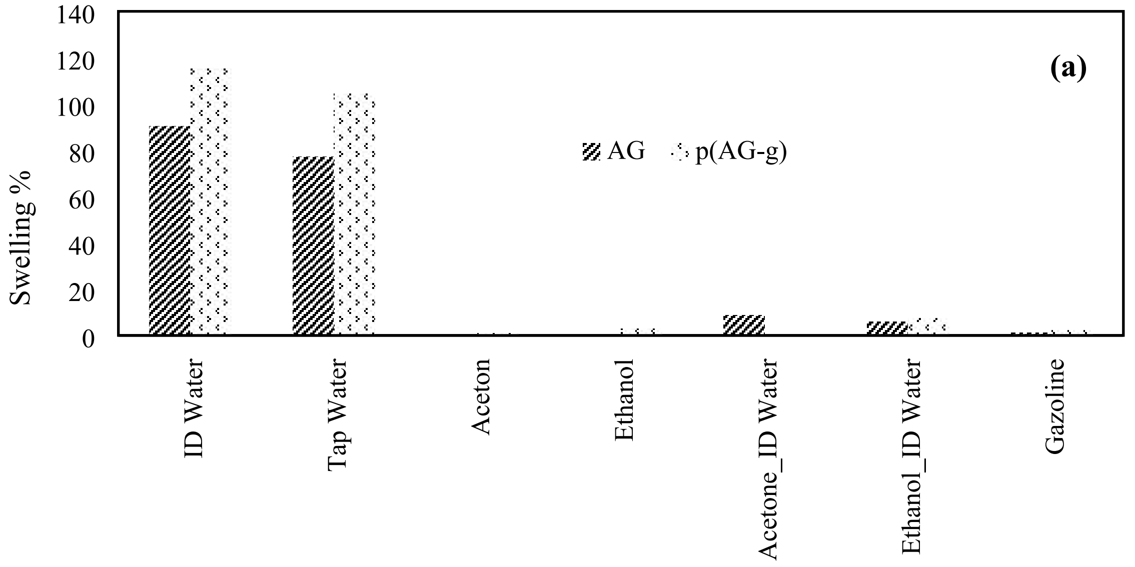

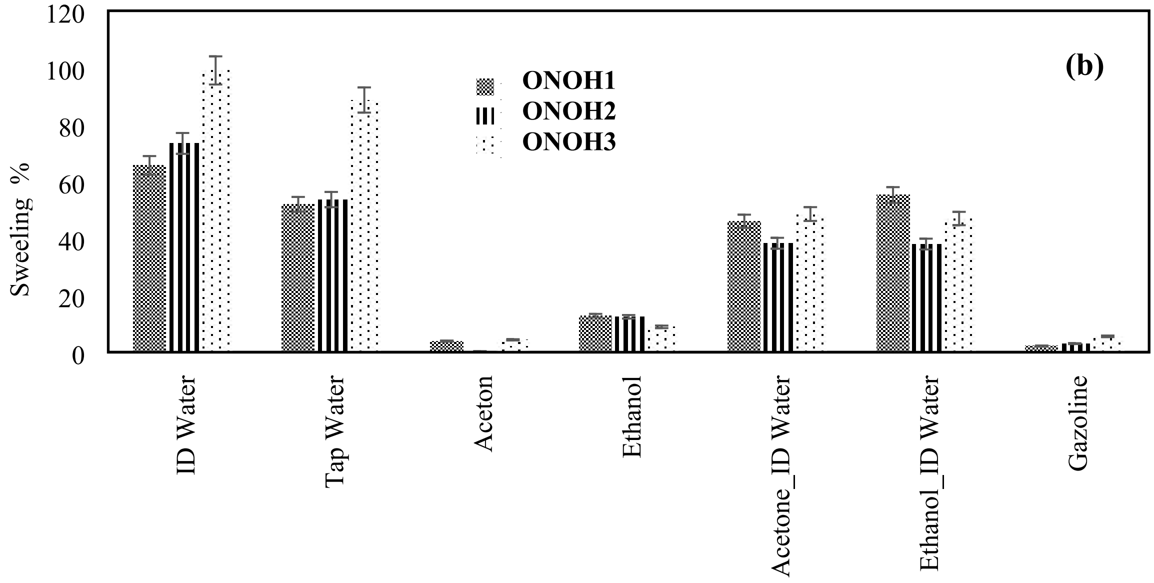

Figure S1. Percent swelling degree of the (a) gel (AG), organohydrogel (p(AG-g)), and (b) ONOHs with time in ID water, tap water, ethanol, acetone, ethanol/ID water (1:1), acetone/ID water (1:1) and gasoline.

Reference

1. D. Alpaslan, T.E. Dudu, N. Aktas, Synthesis and characterization of novel organo-hydrogel based agar, glycerol and peppermint oil as a natural drug carrier/release material, Mater Sci Eng C Mater Biol Appl, 118 (2021) 111534.

2. D. Alpaslan, T. Olak, A. Turan, T. Ersen Dudu, N. Aktas, A garlic oil-based organo-hydrogel for use in pH-sensitive drug release, Chem Zvesti, (2021) 1-14.

3. E. Ömer, D. Alpaslan, T.E. DUDU, H.D. KIVRAK, Novel CA-125 antigen determination in serum by electrochemical methods with onion oil-containing organo-hydrogels, MANAS Journal of Engineering, 11 (2023) 124-135.
